# Supplementary material for: Medical costs of keeping the US economy open during COVID-19
Source: Sci Rep. 2020 Oct 28;10:18422. doi: 10.1038/s41598-020-75280-6 (PMC7595181; doi:10.1038/s41598-020-75280-6)
Supplement: Supplementary file 1 — Supplementary Information. [file 41598_2020_75280_MOESM1_ESM.pdf]

Medical Costs of Keeping the US Economy Open During COVID-19

Jiangzhuo Chen[1], Anil Vullikanti[1,2], Stefan Hoops[1], Henning Mortveit[1,3], Bryan Lewis[1], Srinivasan Venkatramanan[1], Wen You[4], Stephen Eubank[1,4], Madhav Marathe[1,2], Chris Barrett[1,2], and Achla Marathe[1,4,*]

[1]Network Systems Science and Advanced Computing Division, Biocomplexity Institute, University of Virginia, Charlottesville, VA 22904, USA
[2]Department of Computer Science, University of Virginia
[3]Department of Engineering Systems and Environment, University of Virginia
[4]Department of Public Health Sciences, University of Virginia
[*]achla@virginia.edu

ABSTRACT

There is no abstract for this supplementary file.

Appendix: The Disease Model Parameters

The CDC disease model in Figure 1 shows the state transitions which include transmissions and progressions. The former occurs when an individual in *Susceptible* state comes in contact with an individual in one of *Presymptomatic*, *Symptomatic*, or *Asymptomatic* states. The latter occurs when an individual has been in that state for a certain amount of time (called dwell time); the transitions are probabilistic. The dwell time distributions and the transition probability distributions are age dependent and are specified in Table 1.

Progression	Attribute	Age group				
		0-4	5-17	18-49	50-64	65+
Exposed - Asympt	prob	0.35				
Exposed - Asympt	dt-mean	5				
Exposed - Asympt	dt-std dev	1				
Asympt - Recovered	prob	1				
Asympt - Recovered	dt-mean	5				
Asympt - Recovered	dt-std dev	1				
Exposed - Presympt	prob	0.65				
Exposed - Presympt	dt-fixed	1				
Presympt - Sympt	prob	0.65				
Presympt - Sympt	dt-fixed	1				
Sympt - Attd	prob	0.9594	0.9894	0.9594	0.912	0.788
Sympt - Attd	dt-discrete	1:0.175, 2:0.175, 3:0.1, 4:0.1, 5:0.1, 6:0.1, 7:0.1, 8:0.05, 9:0.05, 10:0.05				
Attd - Recovered	prob	1				
Attd - Recovered	dt-mean	5				
Attd - Recovered	dt-std dev	1				
Sympt - Attd(D)	prob	0.0006	0.0006	0.0006	0.003	0.017
Sympt - Attd(D)	dt-fixed	2				
Attd(D) - Hosp(D)	prob	0.95				
Attd(D) - Hosp(D)	dt-fixed	2				
Hosp(D) - Vent(D)	prob	0.06	0.06	0.06	0.15	0.225
Hosp(D) - Vent(D)	dt-fixed	2				
Vent(D) - Death	prob	1				
Vent(D) - Death	dt-fixed	4				
Hosp(D) - Death	prob	0.94	0.94	0.94	0.85	0.775
Hosp(D) - Death	dt-fixed	6				
Attd(D) - Death	prob	0.05				
Attd(D) - Death	dt-fixed	8				
Sympt - Attd(H)	prob	0.04	0.01	0.04	0.085	0.195
Sympt - Attd(H)	dt-fixed	1				
Attd(H) - Hosp	prob	1				
Attd(H) - Hosp	dt-mean	5	5	5	5.3	4.2
Attd(H) - Hosp	dt-std dev	4.6	4.6	4.6	5.2	5.2
Hosp - Recovered	prob,	0.2				
Hosp - Recovered	dt-mean	3.1	3.1	3.1	7.8	6.5
Hosp - Recovered	dt-std dev	3.7	3.7	3.7	6.3	4.9
Hosp - Vent	prob	0.06	0.06	0.06	0.15	0.225
Hosp - Vent	dt-mean	1				
Hosp - Vent	dt-std dev	0.2				
Vent - Recovered	prob	1				
Vent - Recovered	dt-mean	2.1	2.1	2.1	6.8	5.5
Vent - Recovered	dt-std dev	3.7	3.7	3.7	6.3	4.9

Table 1. Age dependent dwell-time distributions and transition probability distributions. Here “dt” refers to duration, e.g. dt-mean is mean duration and dt-discrete is discrete distribution duration. “Attd” is medically attended; “Hosp” is hospitalized; “Vent” is ventilated; “Sympt” is symptomatic and “Asympt” is asymptomatic.
